# Unique intrahepatic transcriptomics profiles discriminate the clinical phases of a chronic HBV infection

**DOI:** 10.1371/journal.pone.0179920

**Published:** 2017-06-29

**Authors:** Jun Hou, Willem P. Brouwer, Kim Kreefft, Lucio Gama, Sarah L. Price, Harry L. A. Janssen, Pim J. French, Thomas Vanwolleghem, Andre Boonstra

**Affiliations:** 1 Department of Gastroenterology and Hepatology, Erasmus MC, University Medical Center Rotterdam, Rotterdam, the Netherlands; 2 Department of Molecular and Comparative Pathobiology, The Johns Hopkins School of Medicine, Baltimore, Maryland, United States of America; 3 Toronto Centre for Liver Disease, University Health Network, Toronto, Canada; 4 Department of Neurology, Erasmus MC, University Medical Center Rotterdam, Rotterdam, the Netherlands; Centre de Recherche en Cancerologie de Lyon, FRANCE

## Abstract

Chronic hepatitis B is a highly heterogeneous liver disease characterized by phases with fluctuations in viral replication and progressive liver damage in some, but not all infected individuals. Despite four decades of research, insight into host determinants underlying these distinct clinical phases—immunotolerant, immune active, inactive carrier, and HBeAg-negative hepatitis–remains elusive. We performed an in-depth transcriptome analysis of archived FFPE liver biopsies of each clinical phase to address host determinants associated with the natural history. Therefore, we determined, for the first time, intrahepatic global expression profiles of well-characterized chronic HBV patients at different clinical phases. Our data, obtained by microarray, demonstrate that B cells and NK/cytotoxic-related genes in the liver, including *CD19*, *TNFRSF13C*, *GZMH*, and *KIR2DS3*, were differentially expressed across the clinical HBV phases, which was confirmed by modular analysis and also Nanostring arrays in an independent cohort. Compared to the immunotolerant phase, 92 genes were differentially expressed in the liver during the immune active phase, 46 in the inactive carrier phase, and 71 in the HBeAg-negative phase. Furthermore, our study also revealed distinctive transcription of genes associated with cell cycle activity, NF-κB signaling, cytotoxic function and mitochondrial respiration between clinical phases. Our data define for the first time using microarray unique transcriptomes in the HBV-infected liver during consecutive clinical phases. We demonstrate that fluctuations of viral loads and liver damage coincide with fluctuations in the liver transcriptome and point to functional- immune and non-immune- components contributing to the clinical phenotype in patients.

## Introduction

Currently about 240 million people are chronically infected with the hepatitis B virus (HBV), and more than 686,000 individuals die annually of HBV-related liver complications such as cirrhosis or hepatocellular carcinoma [1] [2, 3]. The majority of infections occurs prenatally or during childhood, and can persist for decades. During life-long infections, chronic HBV patients progressively go through distinct phases of disease defined by fluctuations in their serum levels of HBV DNA, liver alanine transaminases (ALT) as well as changes in their hepatitis B envelope antigen (HBeAg) status. On the basis of variations in these serum parameters, four clinical HBV phases are defined: the HBeAg-positive immunotolerant (IT) and immune active (IA) phase, and the HBeAg-negative inactive carrier (IC) and HBeAg-negative hepatitis (ENEG) phase. Persistent or intermittent ALT elevations in the IA and ENEG phases reflect hepatic injury due to viral activity and immune activity, and usually require treatment initiation. In contrast, relatively low ALT levels in serum, as observed during the IT and IC phases, suggest minimal disease activity [4, 5].

The value of this clinical distinction has been demonstrated in daily clinical practice for several decades: treatment can generally be withheld in both IT and IC phases, due to a low probability of liver disease progression, but is imperative in the two other phases. However, despite immense efforts in characterizing the host response towards HBV, no definitive intrahepatic immune profile has been found that explains these extreme differences in clinical phases. While we previously demonstrated that the use of systems biology approaches on peripheral blood helps to distinguish the contribution of B cells, NK cells and interferon-stimulated genes (ISG) during the natural history of HBV [6], we now hypothesized that the same technique applied to archived liver biopsy samples could reveal fundamental insights on the intrahepatic host determinants of HBV clinical phases.

Therefore, in the current study, we determined, for the first time, the intrahepatic whole transcriptomes of chronic HBV patients during well-characterized clinical phases. Our study leads to a better understanding of the alterations induced locally as a consequence of prolonged viral infection, which cause fluctuations of HBV DNA and ALT levels in serum and that drive HBV infection towards disease progression and liver damage. We identified that transcriptional activity of B cell-, cytotoxicity/NK cell-, cell cycle-, mitochondrial- and inflammation-associated genes in the liver are distinctive during the HBV clinical phases, while the intrahepatic expression of ISG genes does not correlate with physiological differentiation during the natural course of chronic HBV.

## Methods and materials

### Patients and FFPE liver samples

Core needle liver biopsies of 94 chronic HBV patients with a well-defined clinical phase were collected as part of routine clinical care at the Erasmus MC, and archived as FFPE tissues. Patients were excluded in case they had received antiviral treatment prior to biopsy, or a co-infection with HCV, hepatitis D, hepatitis E or human immunodeficiency virus, presence of auto-immune liver disease, primary biliary cirrhosis, Wilson's disease, hemochromatosis or any other co-existing primary liver disease at the time of biopsy. Histological evaluation was performed as described previously and re-assessed by a single experienced pathologist in a uniform manner [7]. All 94 liver biopsies were transcriptomically profiled, and 52 cases passed the quality control and, in addition were evaluated to have no histological signs of NASH or advanced fibrosis or cirrhosis with a Metavir stage F3/F4. These 52 cases were further analyzed (the core cohort). Sufficient liver tissue was available of 38 cases for immunohistochemical staining for B cells (S1 Table). An independent validation cohort of samples from 24 chronic HBV patients with similar inclusion/exclusion criteria was enrolled in the study to confirm the identified liver transcriptomes (S1 Table). This study was conducted in accordance with the guidelines of the Declaration of Helsinki and the principles of Good Clinical Practice. As the result of the retrospective nature of this study, written informed consent was not obtained from each patient. The ethical review board of the Erasmus Medical Center, Rotterdam, the Netherlands approved of this study as it was in accordance with the FEDERA guidelines, which regulates further use of coded-anonymous residual human tissue for scientific research.

### Definition of chronic HBV clinical phases

Based on serum HBV DNA, ALT levels and HBeAg presence at the time of biopsy, all patients in the study were categorized into four clinical HBV phases according to the EASL guidelines [4] (S1 Table). Immunotolerant (IT) patients had detectable serum HBeAg and repetitive normal ALT values (<40 U/L) for at least 1 year. The HBeAg-positive immune active (IA) and HBeAg-negative (ENEG) patients had repetitive or intermittent abnormal serum ALT (>40 U/L) values, and HBV DNA levels >2,000 IU/mL. Inactive carrier (IC) patients were HBeAg-negative and had both repetitive normal ALT values (<40 IU/L) and HBV DNA levels below 20,000 IU/ mL for at least 1 year.

### DASL microarray processing and quality control

RNA extraction was extracted from FFPE liver samples of chronic HBV patients using the QIAGEN RNeasy FFPE kit, followed by cDNA synthesis and array preparation. The whole genome DASL HT Assay Kit (Illumina) was used to generate biotinylated cDNA. After annealing, extension and ligation, the amplified products were hybridized onto the HumanHT-12v4 BeadChip. The chip was scanned to acquire intensity data, which was further processed using Illumina's GenomeStudio v2010.2 software.

The FFPE liver biopsies used in the current study were archived for 29.3±9.2 years. To control for the quality of transcriptomics profiles, sequential selection was performed based on the overall intensities and the number of detected genes of the array. Arrays for which these 2 parameters fall outside the middle 90% of the distribution range were regarded as quality outliers, and resulted in exclusion of 20 arrays from further analysis (Fig A in S1 File). The average signal of the remaining 52 arrays was 2,300 (SD = 584), with 13,958 (SD = 1,191) genes detected at p-value <0.01 from detection call algorithm integrated in Genomestudio v2010.2 [8]. The expression levels of liver-associated genes TREM1, ALB and ALPL [9] as well as the house-keeping genes GAPDH and ACTB showed consistently high expression across liver FFPE samples. (Fig A in S1 File, and data not shown). The reproducibility of all microarrays of archived FFPE liver samples allowed us to conclude that the intrahepatic gene expression data generated in our study was of high quality (R^2^ = 0.78 based on all reliably detected genes).

### Analysis of gene expression data

Next, the raw data of selected microarrays were read into MATLAB using *ilmnbsread* and subjected to normalization using *quantilenorm* function, which performs sequential quantile normalization, median-polish summarization (first for each gene across all samples, then for each sample across all genes), and then log2 transformation. Non-specific filtering was applied on the normalized data to exclude transcripts with an expression value lower than 3 or with a variance of expression profile less than the 10 percentile. The remaining transcripts (n = 20,818) were annotated with gene names, official symbols, and additional features according to the BeadChip annotation file for HumanHT-12v4 BeadChip, downloaded from the support page at the Illumina web site. In the supervised analyses, each subsequent phase was compared to the IT phase and DEGs were defined as those whose expression levels differed between the reference and any of other phases (IA, IC, or ENEG). To make all clinical phases comparable in respect of the number of samples included, 16 out of 26 cases in the IA phase were randomly selected to perform the supervised analysis. Significance Analysis of Microarray was used to compare expression levels between the reference and other clinical phases on individual genes [10–12], and a 2-fold change and p-value of <0.05 were considered statistically significant as described before [6, 13]. The microarray data has been deposited in NCBI’s Gene Expression Omnibus (GEO) database and is accessible through GEO series accession number GSE83898.

### Modular repertoire analysis

A modular repertoire analysis was conducted using published modules of genes, which were clustered based on similarities in their expression pattern ^30^. The latest version, consisting of 260 modules downloaded using a MATLAB script, was used for the analysis (www.biir.net/public_wikis/module_annotation/Main_Page). To perform liver transcriptomics module analysis, the expression of individual module genes was first extracted from the core cohort. Then a relative transcriptional activity of each module at the IA, the IC and the ENEG phase relative to the IT phase was calculated [6]. This measurement is in units of the standard deviation (SD) and corrected by the size of the given module by a parametric analysis of gene set [14]. The deviations across 3 RTAs from the IA, the IC, and the ENEG phase for individual modules were used to rank all 260 modules. The modules ranked within the upper 10 percentile were selected as the top varied modules (n = 50).

### From liver transcriptomic modules to intrinsic functional clusters

Unsupervised hierarchical ordered partitioning and collapsing hybrid (HOPACH) clustering was used on the top 50 modules to identify intrinsic functional clusters (IFC) which show dynamic modulation during the course of chronic HBV. Nonparametric bootstrapping resampled data in order to determine the most stable clusters [15]. The stability of the clusters was assessed using a similarity measurement between initial clustering and new clustering, and clusters obtained when this iterated measurements reached a maximal value were considered as the most stable clusters [16].

### mRNA quantitation by NanoString

After xylene deparaffinization, total RNA was isolated from FFPE liver biopsies (n = 21) using the RNeasy FFPE Kit (Qiagen) according to the manufacturer’s instructions. The RNA isolation, array preparation, and data processing was performed as described before [17, 18]. A codeset of 526 NanoString fluorescent probes specifically designed for the detection and quantitation of human transcripts associated with immunological pathways was used (nCounter GX human immunology kit).

### Immunohistochemical assessment of B cells

FFPE liver tissue sections from selected patients (n = 38) were stained with antibodies directed against CD19 (Dako) using Ventana BenchMark Ultra stainer following antigen retrieval. The signal was visualized using OptiView DAB IHC Detection Kit (Ventana), and counterstained with hematoxylin. Digital images of 8 randomly selected high-power fields (x20 magnification) within the liver lobuli were captured to count CD19^+^ B cells using NIS-Elements D 3.0 software (Nikon Digital Sight DS-U1). The average number of CD19^+^ cells from 8 high-power fields was determined, and at least 3 portal tracts were counted. The positive surface area was quantified using ImageJ software.

### Statistical analysis on clinical parameters

Statistical analyses were performed with the use of SPSS (v21) software. Baseline variables between cohorts were compared with the use of chi-square tests for categorical variables, or the t-test for continuous variables. All reported P values are two-sided. Significant differences were considered in all cases when p < 0.05.

## Results

### Clinical characteristics of the study population

**Fig 1:**
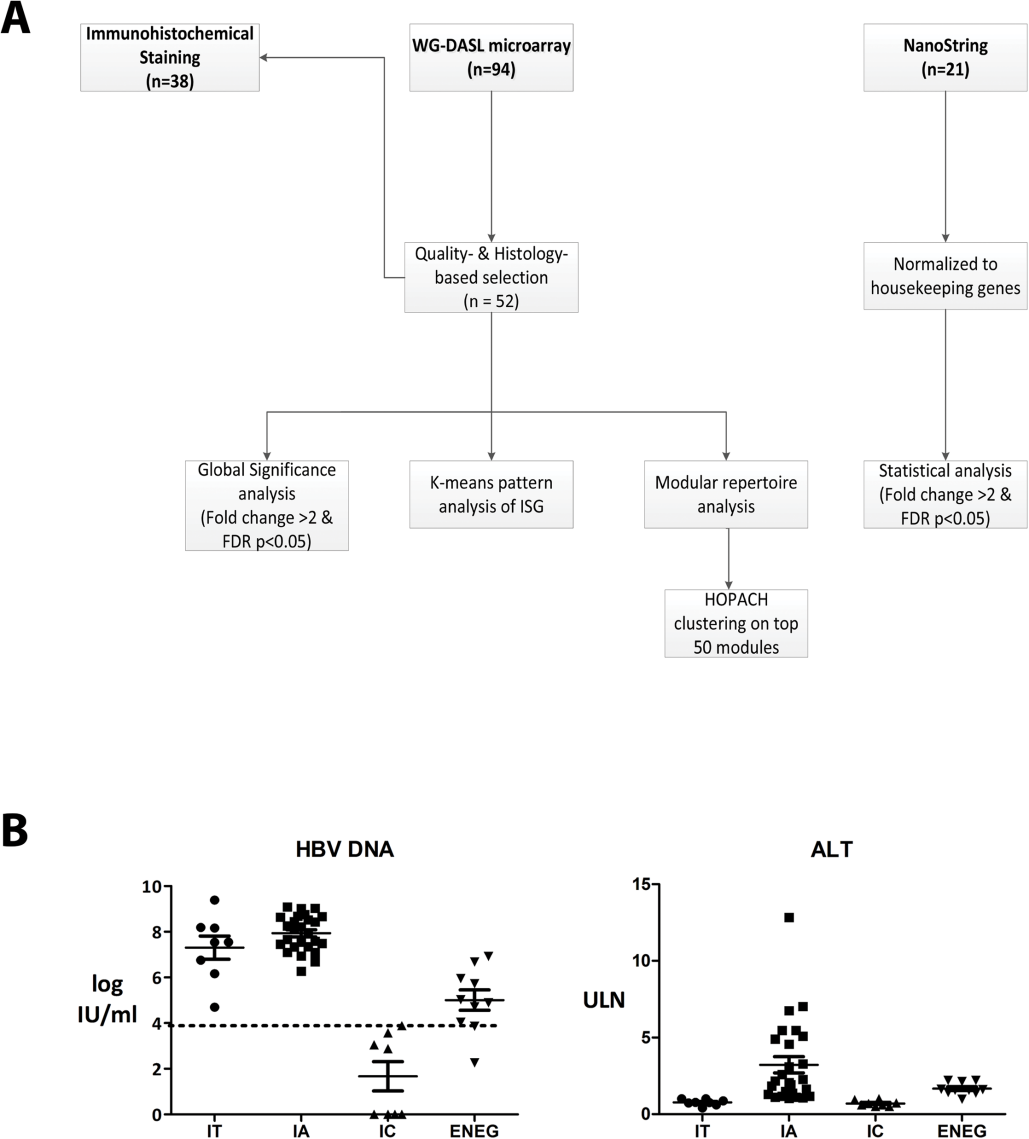
Schematic presentation of patient selection. (A) Total RNA from liver samples of 94 chronic HBV patients were examined by DASL microarray in 2 batches. A quality-based selection excluded 20 arrays from further analysis. Based on HBV DNA and ALT levels, 52 out of 74 cases were further selected as the core cohort, which have a well-defined clinical HBV phase, and a fibrosis grade of 2 or less, and no evidence of NASH or other complications. Significance Analysis of Microarray, K-means pattern analysis, and modular repertoire analysis were performed to get expression signatures of chronic HBV clinical phases. For validating the expression profiling, NanoString was employed to studied the expression of immune genes in an independent liver FFPE cohort and immunohistochemical staining was applied on 38 paired FFPE samples to evaluating the functional status of altered gene expression. (B) Baseline characteristics of chronic HBV patients divided into four clinical phases based on HBV-DNA and ALT levels. ULN: upper limit of normal (40 U/L).

To systematically determine the hepatic gene expression profiles specific for the distinct HBV clinical phase, we first selected formalin-fixed and paraffin-embedded (FFPE) liver biopsies from well-characterized chronic HBV patients, and performed transcriptomics profiling using the WG-DASL array. By applying data quality- and histology-based controls, the profiles of patients with advanced liver fibrosis and NASH were excluded from the analysis (Fig 1A) since we observed that these conditions strongly influenced transcriptomics in liver (data not shown). We selected 52 arrays derived from patients with no concomitant disease and preceded with further analyses. As presented in Fig 1B, high levels of serum HBV DNA and ALT are characteristics of the HBeAg-positive IA and ENEG phases, while the HBeAg-negative IC phase has both low HBV-DNA and ALT levels in serum. The full patient details are presented in S1 Table.

### Numerous genes are differentially expressed in livers of HBV patients in distinct clinical phases

**Fig 2:**
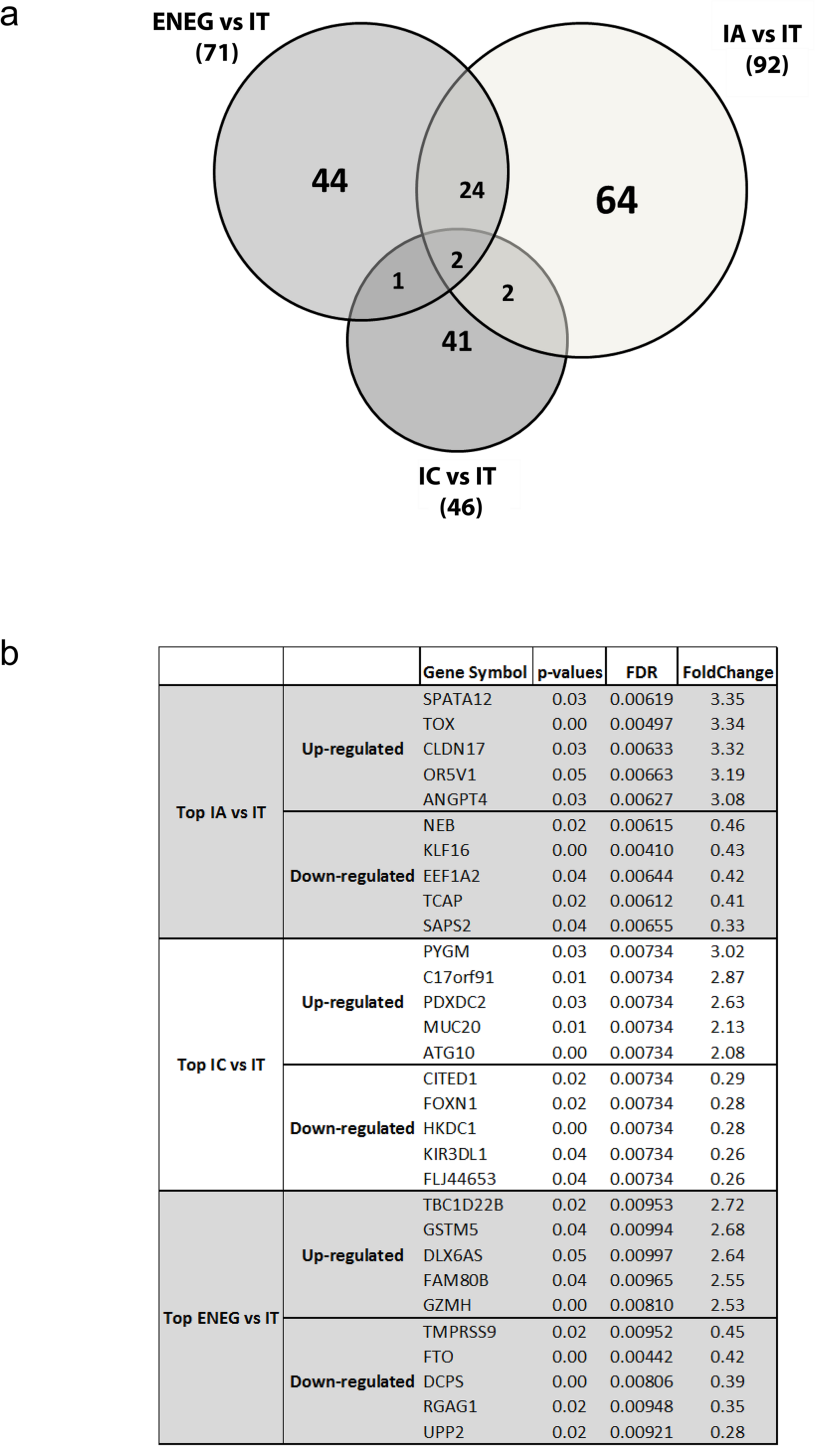
Differentially expressed gene markers for chronic HBV clinical phases. The transcriptomes of clinical HBV phases (IA: 16, IC: 8, ENEG: 10, S1 Table) were compared to the IT (n = 8) phase by a SAM analysis. The SAM analysis defined 178 DEGs between the IT and the subsequent clinical phases at significance level of 2-fold change and p-value < 0.05 (S2 Table). The highest numbers of different transcriptomes were found between the IT and the IA phase (n = 92), followed by the IT and the ENEG phase (n = 71). Forty-six genes were differentially expressed between the IT and the IC phase. (B) The top 5 up- and down-regulated genes and the levels of their expression identified by the SAM analysis of each comparison (IA vs IT, IC vs IT, and ENEG vs IT) are shown in the table.

Microarray analysis of RNA from FFPE-liver biopsies allowed us to compare the hepatic transcriptomes of each clinical phase with the IT phase using Significance Analysis of Microarray (SAM) with a fold-change of 2 and p<0.05 for significance. The IT phase was used as reference since this phase has minimal disease activity and liver pathology, but high serum HBV DNA levels. The analysis showed that the global transcription in HBV infected liver was most distinctive between the IA and the IT phases (n = 92 differentially expressed genes, DEG), followed by the ENEG and the IT phases (n = 71 genes), and the IC and the IT phases (n = 46 genes; Fig 2A and Fig B in S1 File). There was some overlap in DEG between the ENEG and IA phases versus IT, whereas the DEG found in the IC versus IT analysis were mostly unique. A complete list of DEG is shown in S2 Table. The immune related DEG with the most varied expression (top 5 up- or down-regulated) defined by the fold change between the IT and one of the advanced disease phases were *GZMH* and *KIR3DL1* (Fig 2B).

### Intrahepatic ISG gene expression is not correlated with the fluctuation of HBV replication and the extent of liver damage

ISG proteins are potent antiviral effector molecules capable of suppressing viral replication. In blood of chronic HBV patients, we previously observed fluctuating ISG expression levels across the 4 clinical phases, with the highest transcription during the IT phase [6], and demonstrated by qPCR that among blood leukocytes the highest expression of the ISG *CXCL10* is found in monocytes (data not shown). However, in contrast to blood, the DEG in liver material of the 4 clinical phases did not include ISG (S2 Table). To examine this in more detail a pattern analysis for uncovering subgroups was performed on 125 ISG selected from ISG related modules [19], including *CXCL10*, *ISG15*, and *IRF* members. As shown in Fig C in S1 File, similar expression levels in liver for all 125 analyzed ISG genes across the clinical phases were observed irrespective of the number of clusters (i.e. 12, 16, 20, or 24). In an independent cohort separately collected at Erasmus MC using the identical inclusion criteria (n = 21), we validated using NanoString arrays the stable intrahepatic ISG expression across clinical phases of chronic HBV as observed by microarray: the absolute expression levels of most of ISG genes were high (Fig D in S1 File), confirming continuous transcription of ISG in the HBV infected liver that did not correlate with the fluctuations of viral load and ALT levels.

### Intrahepatic CD19^+^ B cells and cytotoxic/NK cells are more pronounced in the immune active phase

**Fig 3:**
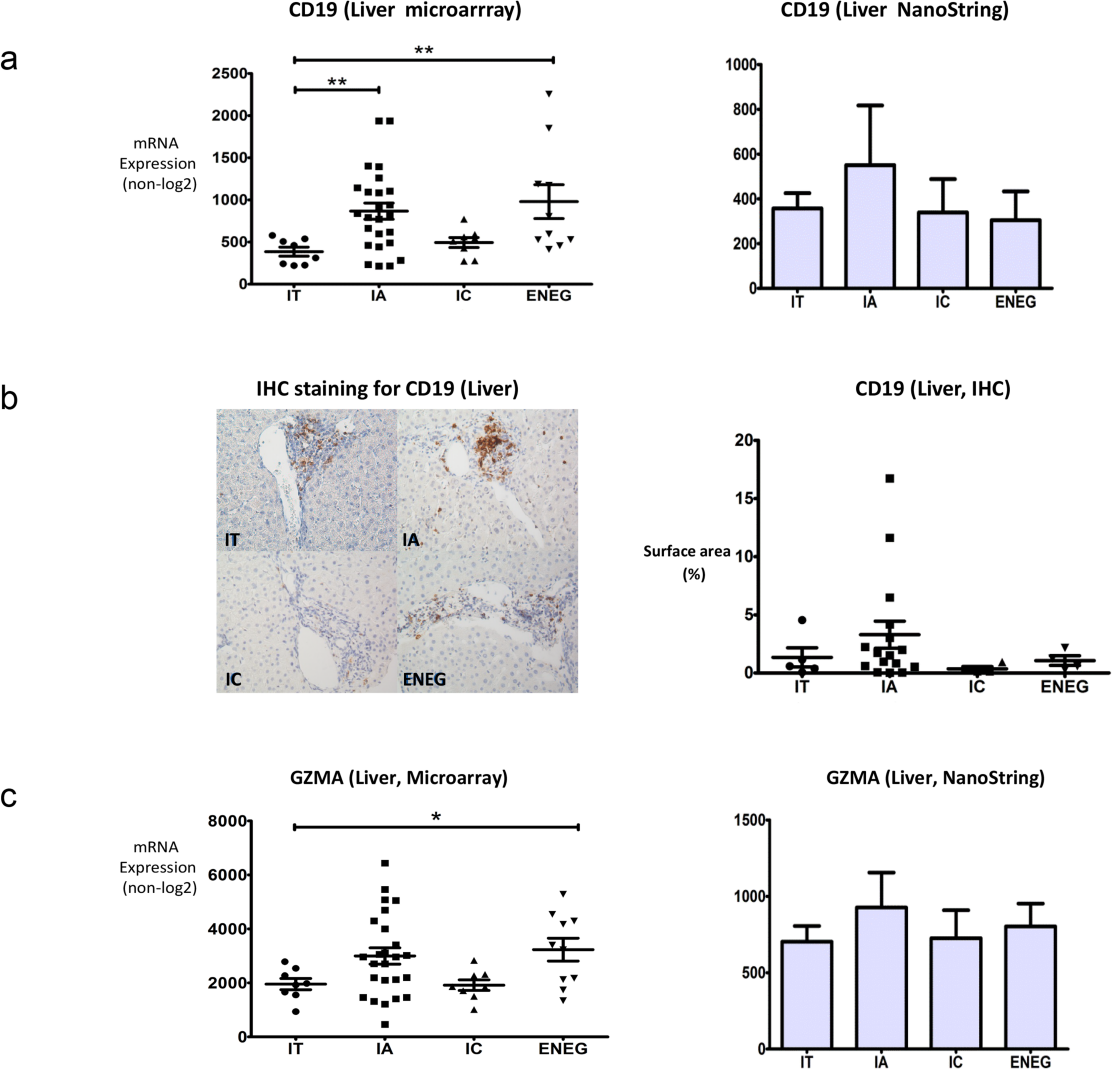
Intrahepatic B cell and cytotoxic/NK cell gene expression is correlated with the fluctuation of HBV replication and ALT levels. (A) The intrahepatic expression of *CD19* mRNA was evaluated by DASL microarray and NanoString assay in an independent cohort. The high transcriptional activities of B cell genes were observed in the IA phase, being the phase that is characterized by relatively high immune activity. (B) The periportal expression of the CD19 protein was detected by immunohistochemistry staining. The staining was quantified using ImageJ software. Highest expression of CD19 was detected in the IA phase. The staining intensity of CD19 in portal tract areas of livers at each clinical phase is shown by the examples in the figure. (C) The intrahepatic expression of *GZMA* was detected by expression microarray and validated in an independent HBV cohort by NanoString assay. The high transcriptional activities of cytotoxic/NK cell genes were observed in the IA phase, being the phase that is characterized by more immune activity. ** denotes p-value < 0.01, * denotes p-value < 0.05.

B cell and cytotoxic/NK cell related genes were previously shown to be differentially expressed in blood of HBV patients at the distinct clinical phases [6], however, it is unclear whether the abundance of these genes is associated with the transition of clinical phases in intrahepatic compartments. Interestingly, *CD19* was among the intrahepatic differentially expressed genes between two or more clinical phases and was relatively high in the IA and ENEG phase as compared to the IT phase. This was confirmed in an independent cohort by Nanostring arrays (Fig 3A and S1 Table).

Immunohistochemical staining for CD19 on liver FFPE tissues obtained from different clinical phases showed that the highest percentage of CD19^+^ cells was found in the portal areas of patients in the IA phase, followed by the ENEG and the IT phases, consistent with the higher transcriptional activity of *CD19* determined by microarray profiling (Fig 3B). The presence of CD19^+^ cells in the lobular areas was relatively low and scattered in all clinical phases (data not shown). Additionally, evaluation of the presence of lymphoid cell infiltration in livers showed that only 4 of the 52 biopsies presented intrahepatic portal follicles, of which 3 were in the IA phase and 1 during the ENEG phase. Histological evaluation of the livers of additional 340 chronic HBV patients from the same tissue bank found that 46 patients exhibited tertiary follicle structures, with 32 (69.5%) at the IA phase, 12 (26.1%) at the ENEG phase, 2 (4.3%) at the IT phase (p<0.05). None of the IC cases were follicle positive. These results strongly indicate that B cell in the IA phase correlate with a higher inflammation status of the liver irrespective of the presence or absence of follicles, which may contribute to histopathological changes.

Among the differentially expressed genes between two or more clinical phases by microarray in the liver was *GZMA*, the gene encoding for granzyme A with higher hepatic expression in the IA (1.58 fold, p = 0.13) and ENEG phase (1.65 fold, p = 0.02)(Fig 3C). Other NK cell/cytotoxic-related DEG included *GZMH and KIR2DS3*, which showed altered expression in the ENEG phase (*GZMH*, *2*.*53 fold*, *p = 0*.*003)* or the IA phase (*KIR2DS3*, *2*.*16 fold*, *p = 0*.*03*) compared to the IT phase (S2 Table). The expression of *GZMH* in the IA and the IT phase did not qualify as a DEG according to our robust definition, however, we noticed that *GZMH had a* 1.99 fold higher expression in the IA compared to the IT (p = 0.03). Similarly, other NK marker genes, including *KIR3DL1*, *KIR2DL4 and KIR2DS5*, were not defined as DEG due to higher p-values, but they showed a considerable increased expression in the IA or the ENEG phase (fold changes ranging between 1.25 and 2.67) while no change (*KIR2DL4*) or a reduction (*KIR3DL1* and *KIR2DS5*) in the IC phase was noted.

### Modular analysis reveals discriminative liver gene transcription across the HBV clinical phases related to B cell, cytotoxicity/NK cell, cell cycle, mitochondrial and inflammation activities

**Fig 4:**
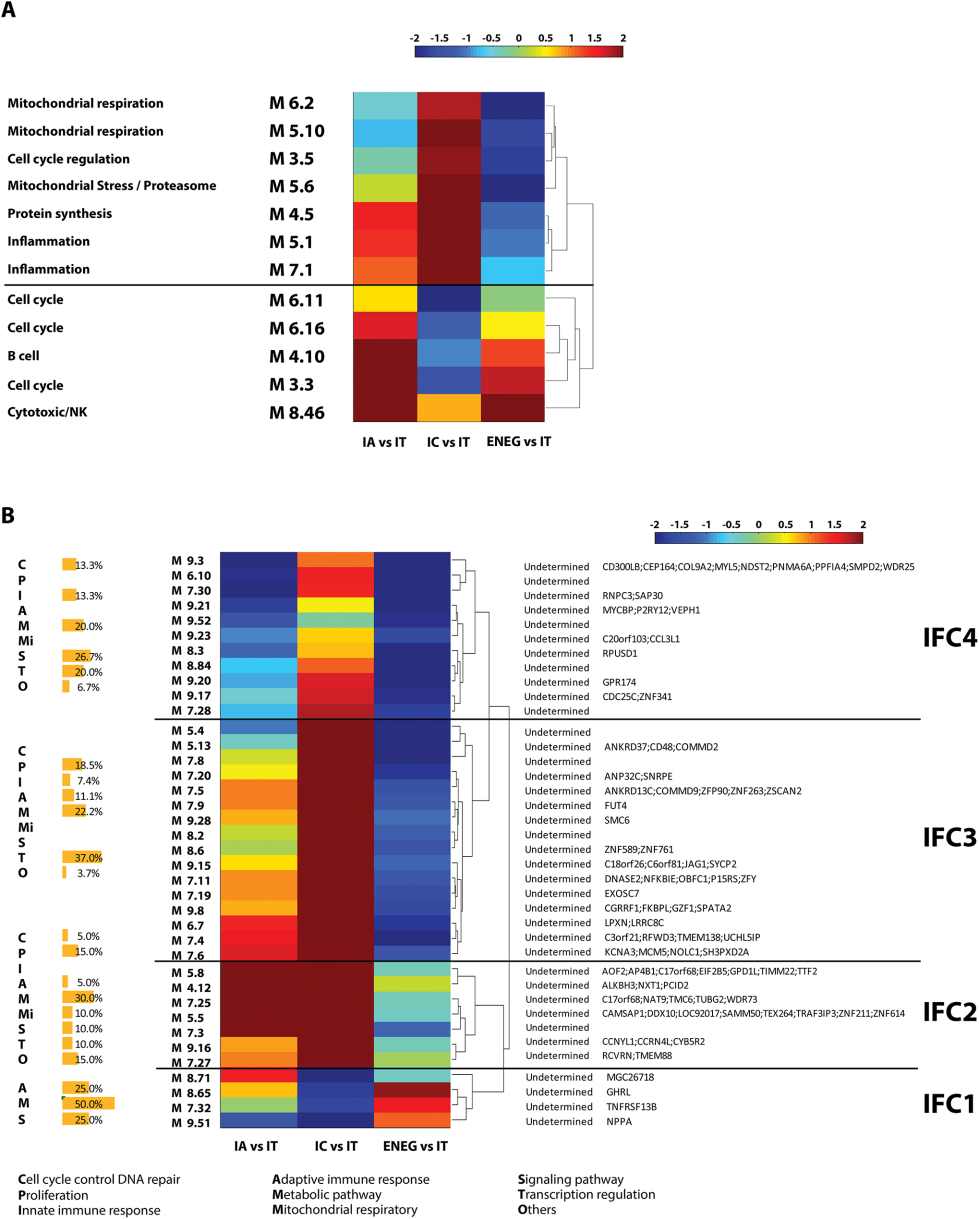
A global module analysis of liver transcriptomics revealed 4 intrinsic functional clusters of HBV clinical phase. (A) An unsupervised clustering was performed on the relative transcriptional activity of 12 functionally determined modules in the core cohort (n = 52), which generated 2 clusters. The clustering using Euclidean distance and ‘complete’ as linkage for the similarity measurement are shown. (B) An unsupervised clustering was performed on the relative transcriptional activity of 38 functionally undetermined modules in the core cohort (n = 52), which generated 4 intrinsic functional clusters (IFC). The functional enrichment of genes in each IFC was described as the percentage of genes with certain function to the total number of genes in that IFC. The member genes, which differed more than 1.5-fold in expression levels between the IT and one of 3 subsequent phases, are listed in the table on the right-hand site.

We further investigated the biological pathways associated with HBV clinical phase, using modular analysis (www.biir.net/public_wikis/module_annotation/Main_Page). The top-50 modules (out of 260) whose transcriptional activities showed the highest variation across the 4 clinical phases represented, amongst others, activities related to B cells, and cytotoxicity/NK cells (Fig 4A and S3 Table), which confirms our findings of differential gene expression in liver obtained from the SAM analysis, highly indicating that B cell function and NK cell function are prominent features of the IA and the ENEG phase ^6^. Also, cell cycle-related modules (M3.3, M3.5, M6.11 and M6.16) were found to have lower activity in the IC phase as compared to the IT phase. The cell division cycle genes *CDC25A* and *CCNB2*, which are included in the cell-cycle modules, showed a more than 1.5 fold change between the IT and IA phase. Also, mitochondrial respiration modules (M5.10 and M6.2) were among the top-50 modules in the liver, which were not observed in blood. Lastly, two modules related to inflammation (M5.1 and M7.1) and one module related to protein synthesis (M4.5) also showed the highest variation in transcriptional activities across the 4 clinical phases.

### Intrinsic functional clusters of chronic HBV clinical phases

Except for the 12 modules described above, the functions of the other 38 modules in the top-50 list that were distinctive among the HBV clinical phases were annotated as “not determined” (www.biir.net/public_wikis/module_annotation/Main_Page). To obtain more information on the immune and biological compartments represented by these undetermined modules, an unsupervised clustering was performed on the 38 functionally undetermined modules based on their relative transcriptional activities. These modules contained 3,289 genes. Of them 91 genes (2.8%) differed more than 1.5-fold in their expression levels between the IT and one of the 3 sequential phases. The unsupervised clustering revealed 4 distinct intrinsic functional clusters (IFC), with IFC1 showing highest activity in ENEG versus IT, IFC2 showing highest activity in IA, and IC versus IT, and IFC3 and IFC4 showing highest activity in IC versus IT (Fig 4B). A knowledge-based gene set enrichment analysis against these genes was used to functionally annotate these IFC (Fig 4B and S3 Table).

There were few genes in IFC1 with an over 1.5-fold change between early and late clinical phases, which included *TNFRSF13B*, a transcript associated with adaptive immune response via stimulating B cells and T cells; and *GHRL* and *CLRN3*, both were involved in metabolic processing. IFC2 was prominent with respect to its high transcription in both the immune active and inactive carrier patients. This cluster was composed of genes associated with metabolic processes (*CYB5R2*, *GPD1L*, *TMC6)*, cell proliferation, (*CCNYL1* and *ZNF211)*, and transcription regulation, (*TTF2* and *ZNF614)*. In addition, there were genes involved in cytoskeleton organization in this cluster and showed over 1.5-fold expression alteration in late phases compared to the immune tolerant phase, including *CAMSAP1*, *RCVRN*, and *TUBG2*. IFC3 and IFC4 showed similar expression patterns and were highly active in the IC compared to the IT phase. They encompassed genes in the IFC3 encoding molecules involved in NF-kB signaling, as evidenced by *NFKBIE*, in cytotoxic function, as *CD48*, *JAG1* and *LPXN*, and in cell proliferation, as *MCM* and *SMC6*. Genes in the IFC4 encoding molecules involved in cell cycle control or innate immunity, such as *CDC25C*, *CEP164*, *CCL3L1*, and *CD300LB*.

## Discussion

In the present study, we profiled the whole transcriptomes of HBV infected livers to determine alterations locally induced by persistent viral infection. We provide direct *in vivo* evidence that transcriptional activity in the liver of B cell genes and cytotoxic/NK cell genes is distinctive during the HBV clinical phases. In contrast, the intrahepatic expression of ISG genes is uncorrelated with physiological differentiation during the natural course of chronic HBV, indicating a unique immune regulatory scenario in liver in response to persistent HBV infection. This was confirmed by various bio-informatics analyses as well as by histology. Furthermore, our study also identified that the transition from the IT to the IA phase was delineated by increased cell cycle activity, the IC phase in the liver was marked by increased activity of NF-κB signaling and cytotoxic function, whereas the ENEG phase showed pronounced expression of genes associated with mitochondrial respiration.

Our findings show hepatic transcriptomes in the liver of chronic HBV patients in the IA and ENEG phase are more activated with respect to the transcription of cytotoxic T cell/NK cell related genes. These include enhanced expression of granzyme genes necessary for target cell lysis, *GZMA*, *GZMB*, and *GZMH* and KIR genes, such as *KIR2DS3*, *KIR3DL1*, *KIR2DL4* and *KIR2DS5* [20–22]. Since the IA and ENEG are characterized by augmented ALT levels as a marker indicating liver inflammation and damage, it is likely that cytotoxic T cell/NK cell activity in the liver may be one of the mechanisms responsible for the enhanced ALT release. Importantly, these findings in the liver are in line with the blood immune fingerprint, which also showed that cytotoxic T cell/NK cell function discriminated between different HBV clinical phases [6]. These findings warrant more mechanistic studies on the phenotype and function of intrahepatic NK cells during disease progression in HBV.

Besides NK cells, active transcription of B cell genes was also observed during the IA and subsequent phases in the liver as compared to the IT phase, including *CD19*, *BLR1*, *BRDG1*, *HLA-DOB*. Abundant B cells in the portal tract areas, and the highest number of tertiary follicles and cellular infiltration was observed in the livers of patients at the IA phase as compared to other phases. Importantly, higher B cell activity at the transcriptional level was also observed in IA samples without obvious tertiary follicles. Again similar as for the cytotoxic/NK cell activity, also enhanced B cell transcriptional activity as a discriminative parameter between different HBV clinical phases was observed in our earlier study in blood [6]. The role of B cells during chronic HBV infection is not well understood. The concordant observations in liver and blood point towards a role for humoral immunity both locally and systemically in the IA phase. Although the potential engagement of humoral immunity in the ENEG phase was not confirmed by immunohistochemistry, a tendency of active involvement of B cells was shown by microarray. However, also antibody-independent functions of B cells, such as a role in antigen presentation, and the regulation of inflammatory responses by the release of the regulatory cytokines IL-10 and IL-35 need to be considered to act during chronic HBV infection [23].

Since ISG exert diverse antiviral effector functions controlling viral replication they would have been likely candidates to influence fluctuations in viral replication rates and ALT levels. However, we show that although ISG are highly expressed in the liver during persistent HBV infection, they are not involved in the transition to the distinct clinical phases since their expression levels remain constant across the phases. In contrast to blood where monocytes were the major source of ISG, immunohistochemical staining for ISG15 showed extensive positivity in all hepatocytes in the HBV infected livers, regardless of clinical phases (data not shown) [24]. This likely explains the discrepancy with our previous observation in blood where a significantly higher expression of ISG was found in the IT phase as compared to subsequent phases [6]. From our data it cannot be concluded that infection of hepatocytes with HBV induces high ISG expression levels since inclusion of healthy livers as reference was not possible due to limited availability of liver samples without pathological indications. Using diseased, non-viral hepatitis liver as reference, such as NASH, complicates the interpretation since these diseases will also induce specific gene profiles. However, it is important to note that in chimpanzees no ISG were induced by HBV in the acute phase of infection [25], whereas intrahepatic ISG expression was induced following HBV infection in woodchucks [26]. During the preparation of this manuscript, Lebossé et al. published a study on intrahepatic innate immunity of untreated HBV patients. Although the patient cohort enrolled in this study is different from our cohort in context of status of fibrosis and intrahepatic inflammation, as well as the distribution of clinical phases, the authors obtained similar observations: the expression of ISG has no or weak correlation with serum or intrahepatic levels of HBV DNA and ALT [27].

HBV replicates in hepatocytes in the liver. These cells regulate a wide variety of biochemical reactions, including the storage, synthesis and breakdown of diverse molecules, such as vitamins, hormones and proteins. With this in mind, we found that the distinctive hepatic gene profiles of HBV clinical phases not only included immune modalities, but also genes related to mitochondrial function (increased during the ENEG phase), altered cell cycle regulation (increased during the IA phase), and highly transcribed genes associated with lipid metabolism during the IC phase. An association between HBV replication and dysregulated cell cycle in HBV infected hepatocytes has been suggested by in vitro studies, which have shown that the HBV X protein (HBx) modulates the regulation of cell cycle and hence promotes cell proliferation, cell growth, cell survival of infected hepatocytes. HBx enhances cell viability via transactivating various cell cycle regulators, such as cyclins and cell cycle inhibitors [28–30]. A recent whole transcriptome study provides further evidence that HBV infection alters the expression of a number of genes associated with cell cycle regulation [31]. These results are consistent with what we observed in transcriptomes of HBV infected liver. Our liver transcriptomics data revealed a higher activity of cell cycle regulation in the IA phase -which is characterized by high rates of viral replication- with upregulated expression of *CDC20*, *CDC25A*, *CCNB2*, *CCNYL1*, *CEP164* and other genes associated with cell cycle regulation. Although we cannot exclude that non-hepatocytes also exhibit modulation of the levels of cell-cycle genes, our data provides direct *in vivo* evidence to support the correlation between the IA phase and cell cycle arrest, and suggests that cell cycle progression is a distinctive process in the events taking place during the natural history of chronic HBV. Moreover, we also observed an increased mitochondrial effect in the ENEG phase. The functional role of mitochondria in chronic HBV clinical phases is not well established, but a recent study showed evidence of co-localization of HBx and the mitochondrial membrane protein COXIII. As a result of the co-localization, mitochondrial function and reactive oxygen species were upregulated [32]. Interesting, our own observation following metabolomic profiling of serum of chronic HBV patients, pointed out that hijacking of the G3P–NADH shuttle that results from altered mitochondrial membrane transportation is prominent in the IC and ENEG phases of HBV infection [33]. These observations strongly suggest a link between HBV replication and induced mitochondrial respiratory activity.

In conclusion, profiling of liver transcriptomes of chronic HBV patients during distinct clinical phases indicates that intrahepatic expression patterns of anti-HBV immune components are largely mirrored by the expression profiles of their counterparts in blood, especially for B cell and cytotoxicity/NK cell activity. Moreover, on the basis of gene expression profiling of the liver the contribution of ISG to the natural history of HBV appears to be limited. In contrast, important differences were observed between the liver and blood in transcription levels of cell cycle and mitochondrial genes between the different clinical HBV phases. The identification of these intrahepatic gene expression profiles for the specific immune functions and cellular processes associated with HBV clinical phases, warrant more mechanistic and functional studies to determine their role in disease progression in chronic HBV patients.

## Supporting information

S1 File **Fig A in S1 File. DASL Microarray processing and data analysis.** Out of 94 profiled FFPE liver biopsies by Whole-Genome DASL Assay, 74 were selected sequentially based on the overall intensities, as well as the number of detected genes, which needs to fall within the middle 90% of the distribution range. The remaining 74 arrays were further processed in the MATLAB using ilmnbsread and normalized using quantilenorm function, which performs sequential quantile normalization, median-polish summarization (first for each gene across all samples, then for each sample across all genes), and then log 2 transformation. Non-specific filtering excluded transcripts with low expression or small variability across all samples and resulted in 20,818 transcripts for the downstream analyses. **B.** The expression of liver specific gene TF in each sample was evaluated in comparison with housekeeping gene GAPDH and blood specific gene TREM1 to preclude potential problems arising from degraded tissues and blood contamination. As shown in the figure, the expression of TF is constantly high across all FFPE liver tissues (on X-axis) enrolled in the core cohort (n = 52), comparable to GAPDH. In contrast, the blood specific gene TREM1 has low expression in the same FFPE liver tissues. The X-axis shows individual patients. The complete expression data is accessible through NCBI’s GEO database. **Fig B in S1 File. Differentially expressed genes in advanced clinical phases compared to the IT phase. A.** A heatmap showing 177 unique genes differentially expression in advanced clinical phases compared to the IT phase in the core cohort (n = 52) (see Method section. The clustering was performed using Euclidean distance and ‘complete’ as linkage for the similarity measurement. **B.** The expression of CD79B and BCL2 (B cell related genes), CCR6 and TNFRSF13C (ISG), ADA and BATF3 (identified DEG) was validated in an independent cohort by NanoString. **Fig C in S1 File. Intrahepatic ISG gene expression is not correlated with the fluctuation of HBV replication and ALT levels.** To evaluate the dynamics of ISG expression in liver of chronic HBV patients, a pattern analysis was performed in order to define ISG subgroups, which are differentially expressed between distinct clinical phases. ISG were extracted from 3 interferon related transcriptomics modules (http://www.biir.net/public_wikis/module_annotation/V2_Trial_8_Modules). The pattern analysis was performed on the core cohort (n = 52) for 12, 16, 20 or 24 subgroups. All analyzed ISG (n = 137) presented similar expression levels across the clinical phases, indicating no correlation between ISG expression and chronic HBV progression in liver. The pattern analysis with 16 subgroups is shown in the figure. **Fig D in S1 File. Intrahepatic ISG gene expression is not correlated with the fluctuation of HBV replication and ALT levels.** The expression patterns of 26 ISG in an independent cohort of 21 chronic HBV patients were evaluated by NanoString. The heatmap shows the expression levels of individual genes (rows) in 21 patients (columns). The black lines separate clinical phases. The intensity values are normalized to the median of a specific gene across all patients. Red represents relatively high expression levels, and blue represents relatively low levels. The analyzed ISG presented similar expression levels across all clinical phases, including CXCL10, TNFRSF10, and IRF members, indicating some variation, but no correlation between ISG expression and HBV clinical phases in livers.
(PDF)

S1 Table Clinical characteristics of enrolled patients in the study. (PDF)

S2 Table Gene markers for HBV clinical phases identified by a SAM analysis with a significant cutoff of 2 fold change (both up- and down-regulated) and p-value of 0.05 compared to the IT as reference. (PDF)

S3 Table The top varied modules with determined function were clustered into 2 clusters based on their transcriptional activity relative to the IT phase, which had distinct activity patterns in HBV clinical phases. (PDF)
