# Supplementary material for: Unique intrahepatic transcriptomics profiles discriminate the clinical phases of a chronic HBV infection
Source: PLoS One. 2017 Jun 29;12(6):e0179920. doi: 10.1371/journal.pone.0179920 (PMC5491066; doi:10.1371/journal.pone.0179920)
Supplement: S1 File — Fig A in S1 File. DASL Microarray processing and data analysis. Out of 94 profiled FFPE liver biopsies by Whole-Genome DASL Assay, 74 were selected sequentially based on the overall intensities, as well as the number of detected genes, which needs to fall within the middle 90% of the distribution range. The remaining 74 arrays were further processed in the MATLAB using ilmnbsread and normalized using quantilenorm function, which performs sequential quantile normalization, median-polish summarization (first for each gene across all samples, then for each sample across all genes), and then log 2 transformation. Non-specific filtering excluded transcripts with low expression or small variability across all samples and resulted in 20,818 transcripts for the downstream analyses. B. The expression of liver specific gene TF in each sample was evaluated in comparison with housekeeping gene GAPDH and blood specific gene TREM1 to preclude potential problems arising from degraded tissues and blood contamination. As shown in the figure, the expression of TF is constantly high across all FFPE liver tissues (on X-axis) enrolled in the core cohort (n = 52), comparable to GAPDH. In contrast, the blood specific gene TREM1 has low expression in the same FFPE liver tissues. The X-axis shows individual patients. The complete expression data is accessible through NCBI’s GEO database. Fig B in S1 File. Differentially expressed genes in advanced clinical phases compared to the IT phase. A. A heatmap showing 177 unique genes differentially expression in advanced clinical phases compared to the IT phase in the core cohort (n = 52) (see Method section. The clustering was performed using Euclidean distance and ‘complete’ as linkage for the similarity measurement. B. The expression of CD79B and BCL2 (B cell related genes), CCR6 and TNFRSF13C (ISG), ADA and BATF3 (identified DEG) was validated in an independent cohort by NanoString. Fig C in S1 File. Intrahepatic ISG gene expression is not corr [file pone.0179920.s001.pdf]

Supplement to Material and Methods section:

RNA extraction and cDNA synthesis

RNA was extracted from FFPE liver samples of chronic HBV patients, and RNA was purified using the QIAGEN RNeasy FFPE Kit following the protocol provided by the manufacturer. In detail, paraffin was removed from FFPE tissue sections by treatment with xylene, followed by ethanol-mediated extraction of residual xylene. Next, samples were treated with an optimized buffer containing proteinase K to de-crosslink RNA and cellular proteins, and release RNA, followed by DNase treatment and RNA purification using RNeasy MinElute spin columns.

The concentration of the RNA samples was measured with RiboGreen. The required concentration is set to 40 ng/μl. To obtain a relative measure of RNA quality prior to Whole Genome DASL Assay analysis, samples were analyzed by qPCR of actin mRNA.

Total RNA (>100 ng) was converted to cDNA using both biotinylated random nonamers and biotinylated oligodT using the Whole Genome DASL HT Assay Kit (Illumina). Probe groups are annealed to the biotinylated cDNA, followed by selection of the duplexes on streptavidin beads to remove unhybridised oligos. Correctly annealed, assay-specific oligos were extended and ligated to a locus-specific oligo. The locus-specific oligo incorporated an address sequence and primer site for the generation of amplifiable products. Templates were labeled during PCR amplification and subsequently hybridized onto the HumanHT-12v4 BeadChip. The array was scanned to acquired intensity data. The entire experimental procedure was carried out according to Illumina protocols.

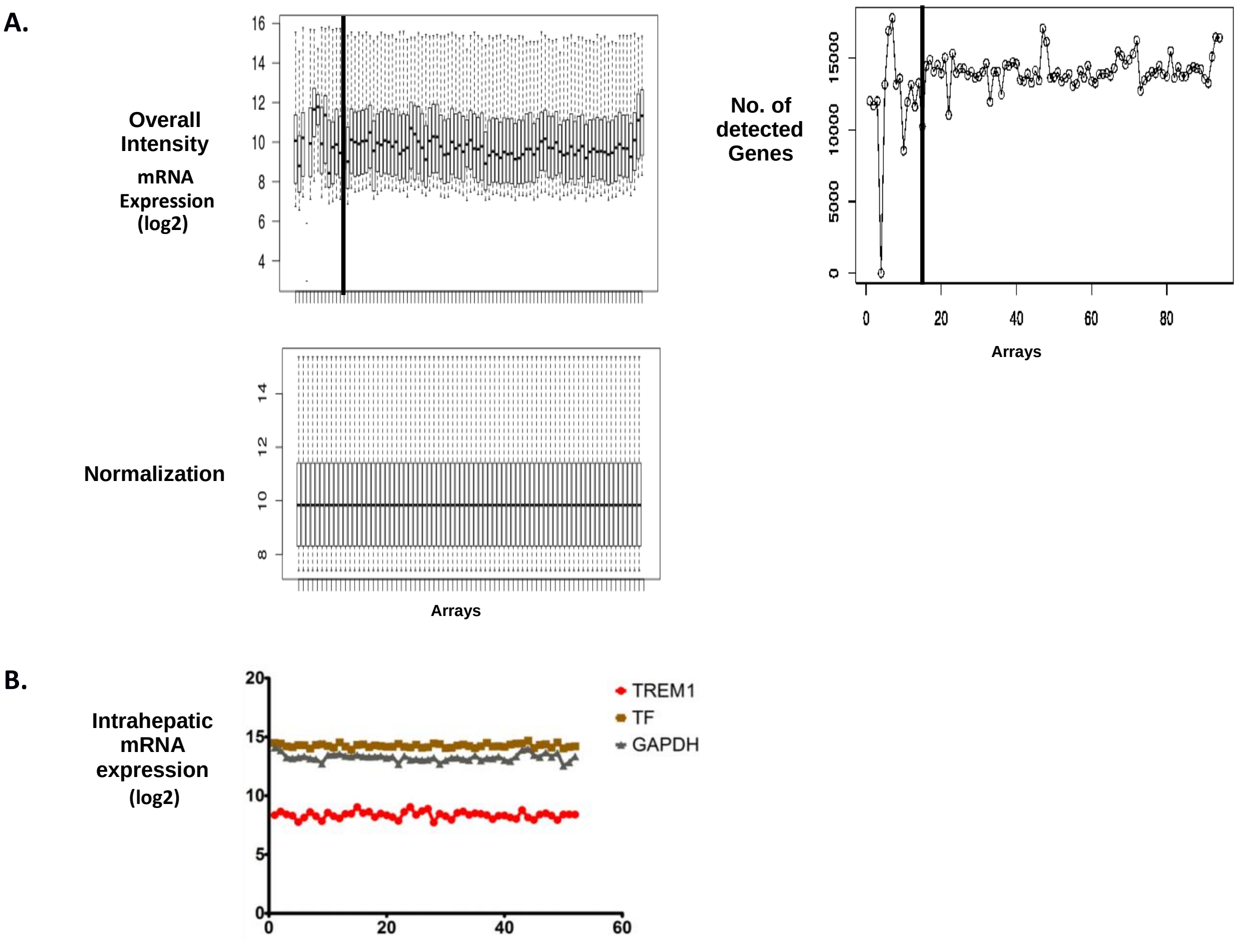

Fig A: DASL Microarray processing and data analysis

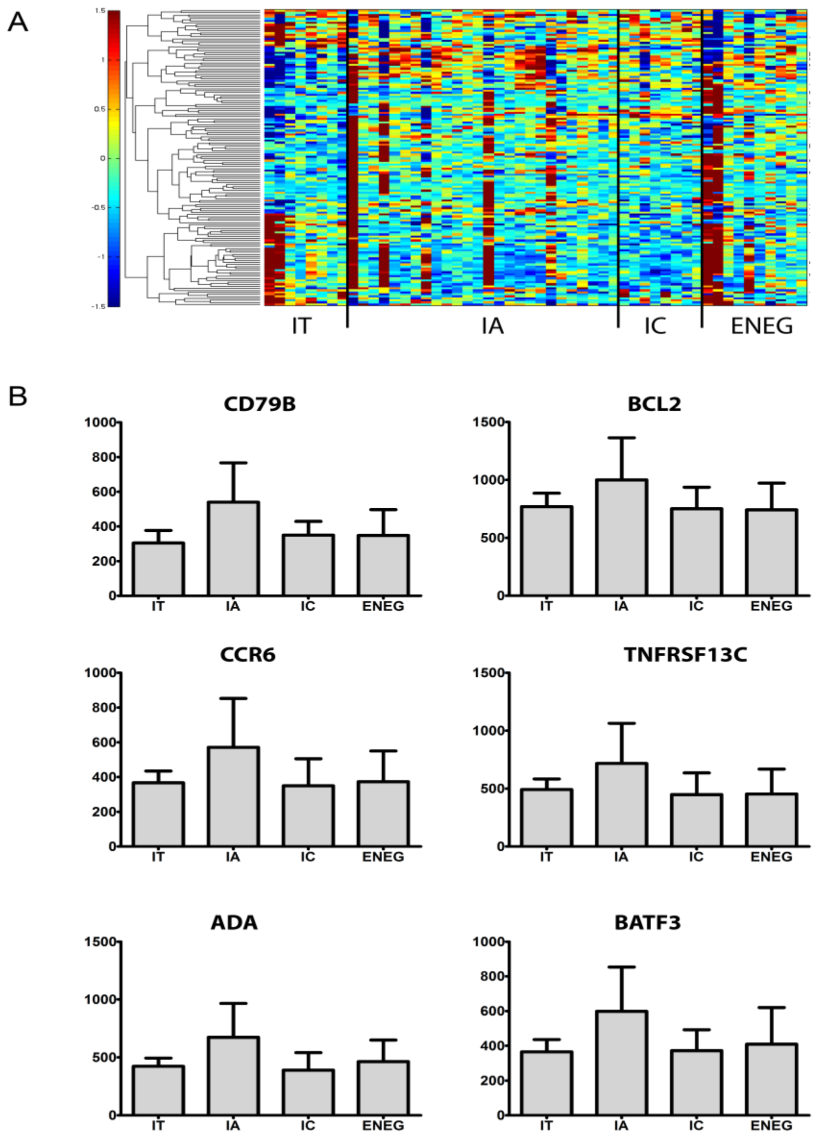

Fig B: **Differentially expressed genes in advanced clinical phases compared to the IT phase**

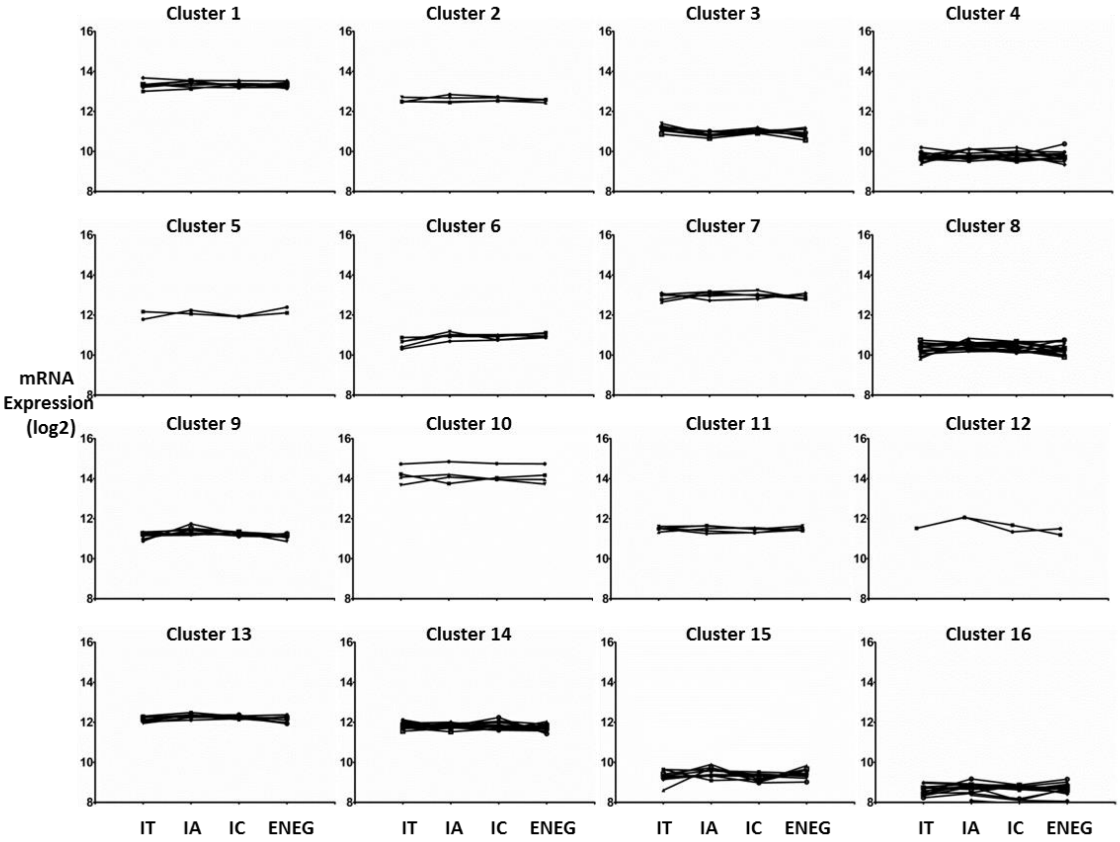

cluster	
1	ABCA1, ACTA2, ECGF1, IFITM3, PARP10, STAT1, XAF1
2	FBXO6, MOV10, RBCK1, TRIM56
3	DYNLT1, GBP1, IFIT3, LAP3, LGALS3BP, OAS3, PLSCR1, TRIM5
4	C1QA, CPT1B, GBP5, LHFPL2, MT1A, NTNG2, OASL, PML, RSAD2, SAMD9L, TDRD7, UNC93B1
5	TAP1, TMEM140
6	NCOA7, SAMD9, SAMD9, SP100, SRBD1, TRIM22
7	DRAP1, GBP2, HERC5, IFITM1, LY6E
8	AIM2, BST2, BTN3A1, HERC6, IFIH1, IFIT2, ISG15, LGALS9, NBN, NTSC3, PARP14, PSMB9, REC8, TAP2, ZNF684
9	GBP3, IRF7, PARP12, PARP9, RHBDF2, TRIM38, WARS
10	EIF2AK2, MT2A, MX1, STAT2
11	IFI16, OAS1, PHF11, SP110, TRIM25
12	CXCL10, PRIC285
13	ADAR, HES4, IFI35, IFI44L, ISG20, KIAA1618, OAS2, SEPT4, TIMM10
14	APOL6, CEACAM1, CHMP5, DHX58, EPSTI1, GADD45B, GALM, GBP4, IFI44, IFIT1, SCO2, SERPING1, TNFSF10, TRIM21, UBE2L6, ZC3HAV1
15	BATF2, CASP1, CCL8, NMI, RTP4, SOCS1, SP140, TCN2, TRAFD1, ZBP1
16	ATF3, DDX58, DHRS9, ETV7, GBP6, HSH2D, IFIT5, INDO, LAMP3, MDK, OTOF, TNFAIP6, TRIM6, ZNFX1

Fig C: **Intrahepatic ISG gene expression is not correlated with the fluctuation of HBV replication and ALT levels**

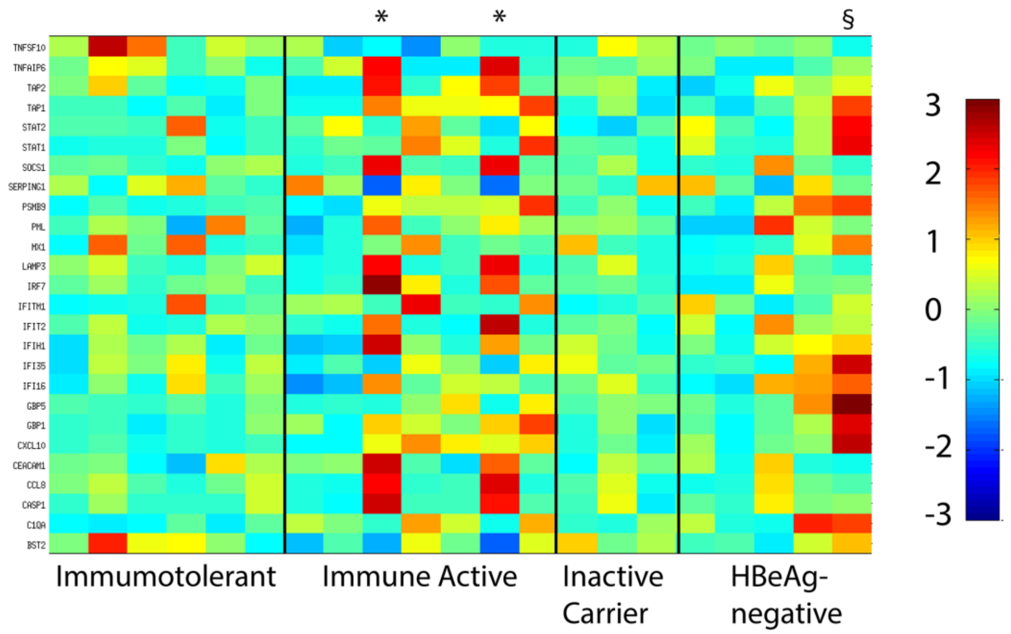

* HBV genotype: C
§: ALT: 236(IU/l)

Fig D: Intrahepatic ISG gene expression is not correlated with the fluctuation of HBV replication and ALT levels
