# Supplementary material for: Unique intrahepatic transcriptomics profiles discriminate the clinical phases of a chronic HBV infection
Source: PLoS One. 2017 Jun 29;12(6):e0179920. doi: 10.1371/journal.pone.0179920 (PMC5491066; doi:10.1371/journal.pone.0179920)
Supplement: S1 Table — (PDF) [file pone.0179920.s002.pdf]

-

S1 Table. Clinical characteristics of enrolled patients in the study

	Immune tolerant	HBeAg-positive active hepatitis	Inactive carrier	HBeAg-negative active hepatitis
**WG-DASL cohort**	**(N=8)**	**(N=26)**	**(N=8)**	**(N=10)**
**Demography**				
Age at biopsy, year (SD)	25.5 (2.3)	27.0 (1.7)	31.5 (3.0)	35.9 (3.2)
Female, n (%)	7 (13%)	18 (35%)	7 (13%)	3 (6%)
Body mass index, kg/m2 (SD)	21.7 (0.7)	22.5 (0.6)	22.2 (1.1)	22.1 (1.1)
**Race, n (%)**				
Asian	7 (88%)	19 (73%)	2 (25%)	7 (70%)
Caucasian	1 (13%)	5 (19%)	6 (75%)	1 (10%)
Other	0 (0%)	2 (8%)	0 (0%)	2 (20%)
**Virology**				
log HBV DNA, IU/ml (SD)	7.3 (0.5)	7.9 (0.2)	1.7 (0.6)	5.0 (0.4)
HBV genotype A/B/C/D/E	0/3/3/1/0*	1/8/11/4/1*	0/1/1/6/0	1/2/4/3/0
**Chemistry/hematol**				
ALT, x ULN (SD)	0.8 (0.1)	3.2 (0.5)	0.7 (0.1)	1.7 (0.1)
**Histology**				
Median Ishak fibrosis, (IQR)	0 (0.0-2.5)	1.5 (0.0-3.0)	0 (0.0-0.0)	2.5 (1.3-3.0)
Hepatic Activity index, (SD)	2.3 (0.7)	4.2 (0.6)	1.5 (0.2)	4.0 (0.5)

**IHC cohort**	**(N=6)**	**(N=19)**	**(N=6)**	**(N=7)**
**Demography**				
Age at biopsy, year (SD)	25.17 (6.85)	25.53 (7.73)	32.00 (9.03)	35.00 (10.66)
Female, n (%)	5 (83.3%)	13 (68.4%)	5 (83.3%)	3 (42.9%)
Body mass index, kg/m2 (SD)	21.34 (2.25)	22.45 (2.88)	21.75 (2.94)	22.26 (3.72)
**Race, n (%)**				
Asian	5 (83.3%)	14 (73.7%)	2 (33.3%)	5 (71.4%)
Caucasian	1 (16.7%)	4 (21.1%)	4 (66.7%)	1 (14.3%)
Other	0 (0.0%)	1 (5.3%)	0 (0.0%)	1 (14.3%)
**Virology**				
log HBV DNA, IU/ml (SD) **δ**	7.3 (1.6)	8.2 (0.6)	2.2 (1.8)	4.6 (1.6)
HBV genotype A/B/C/D/E	0/3/2/1/0	0/7/7/3/1*	0/1/1/4/0	1/1/3/2/0
**Chemistry/hematol**				
ALT, x ULN (SD) **δ**	0.8 (0.2)	3.7 (3.0)	0.7 (0.2)	1.7 (0.3)
**Histology**				
Median Ishak fibrosis, (IQR) **δ**	0.5 (0.0-3.0)	2.0 (0.0-3.0)	0 (0.0-0.0)	2.0 (1.0-3.0)
Hepatic Activity index, (SD) **δ**	2.50 (1.87)	4.58 (3.11)	1.33 (0.52)	4.00 (2.00)

**Nanostring cohort**	**(N=6)**	**(N=7)**	**(N=3)**	**(N=5)**
**Demography**				
Age at biopsy, year (SD)	33.17 (7.83)	28.14 (9.10)	40.17 (9.54)	34.00 (10.68)
Female, n (%) **$**	5 (83.3%)	1 (14.3%)	3 (100.0%)	1 (20.0%)
Body mass index, kg/m2 (SD)	23.21 (3.54)	22.78 (2.98)	23.53 (2.12)	24.95 (3.75)
**Race, n (%)**				
Asian	5 (83.3%)	5 (71.4%)	1 (16.7%)	2 (40.0%)
African	0 (0.0%)	0 (0.0%)	0 (0.0%)	1 (20.0%)
Other	1 (16.7%)	2 (28.6%)	2 (33.3%)	2 (40.0%)
**Virology**				
log HBV DNA, IU/ml (SD) **$**	8.79 (0.38)	7.66 (2.30)	3.07 (1.10)	3.92 (1.53)
HBV genotype A/B/C/D/E **$**	0/4/1/1/0	1/1/4/1/0	0/0/1/2/0	0/0/0/5/0
**Chemistry/hematol**				
ALT, x ULN (SD) **$**	0.6 (0.2	1.5 (0.5)	0.6 (0.2)	2.6 (1.9)
**Histology**				
Median ishak fibrosis, (IQR) **$**	1.0 (0.8-1.0)	2.0 (1.0-2.0)	0 (0.0-0.0)	2.0 (1.0-2.0)

δ: $p < 0.05$ for test between clinical phases
$: $p < 0.05$ for test between clinical phases
*: 1 unknown
