# Supplementary material for: Unique intrahepatic transcriptomics profiles discriminate the clinical phases of a chronic HBV infection
Source: PLoS One. 2017 Jun 29;12(6):e0179920. doi: 10.1371/journal.pone.0179920 (PMC5491066; doi:10.1371/journal.pone.0179920)
Supplement: S2 Table — (PDF) [file pone.0179920.s003.pdf]

-

S2 Table. Gene markers for HBV clinical phases identified by a SAM analysis with a significance cutoff of 2 fold change (both up- and down-regulated) and p-value of 0.05 compared to the IT as reference

*** FC = Fold Change (both up- and down-regulated), DEG = Differentially Expressed Gene, q-values = Estimated false positive by Significance Analysis of Microarrays**

IA versus IT (92 DEG)	p-values	q-values	FC
ANGPT4	0.03	0.239	3.08
ARHGAP11B	0.02	0.073	2.15
AURKA	0.01	0.103	2.04
BATF3	0.01	0.007	2.27
C12orf48	0.02	0.065	2.13
C12orf49	0.01	0.007	2.45
C1orf75	0.03	0.073	2.41
C5orf46	0.03	0.239	2.10
CA12	0.03	0.087	0.49
CCL3L1	0.02	0.268	2.68
CLDN17	0.03	0.220	3.32
CXCL9	0.00	0.052	2.29
DPEP3	0.01	0.007	2.41
EEF1A2	0.04	0.103	0.42
EFNA5	0.01	0.120	2.60
FABP3	0.00	0.036	2.08
FAM111B	0.03	0.087	2.29
FAM159A	0.03	0.288	2.20
FAM179A	0.00	0.016	2.68
FAM74A3	0.05	0.239	2.05
FLJ25328	0.02	0.239	2.00
FOXD4L5	0.01	0.220	2.44
GINS2	0.02	0.016	2.23
GINS3	0.03	0.087	2.17
GLI1	0.02	0.120	2.18
GNA15	0.02	0.027	2.05
GPR114	0.00	0.000	2.06
HAS1	0.01	0.120	2.21
HERC2P4	0.03	0.147	2.42
HOXA13	0.02	0.268	2.87
INSL3	0.03	0.196	2.02
KIR2DS3	0.03	0.196	2.16
KLF16	0.00	0.040	0.43
KRT72	0.05	0.288	2.45
LINCR	0.02	0.129	2.22
LOC222967	0.03	0.103	2.03
MCOLN2	0.00	0.016	2.14
MIR181B1	0.02	0.120	2.96
MIR2276	0.04	0.040	0.48
MUSK	0.05	0.268	2.20
MYBPH	0.04	0.147	2.44
MYLK4	0.01	0.052	2.29
NEB	0.02	0.045	0.46
OR5H15	0.04	0.312	2.82
OR5V1	0.05	0.288	3.19
PLAC8L1	0.02	0.147	2.59
PRPH	0.01	0.045	2.08
PTCHD1	0.05	0.220	2.23
RPS21	0.01	0.052	0.48
SAPS2	0.04	0.045	0.33
SHH	0.02	0.040	0.48
SLC22A4	0.03	0.326	2.10
SLC39A2	0.04	0.268	2.35
SPATA12	0.03	0.171	3.35
TCAP	0.02	0.040	0.41
TNFRSF13C	0.01	0.073	2.48
TOX	0.00	0.023	3.34
UBD	0.02	0.040	2.04
UCP1	0.04	0.326	2.22
UHRF1	0.01	0.065	2.20
VTCN1	0.00	0.000	2.14
XKRY	0.03	0.312	2.36
ZC3H12D	0.00	0.007	2.26
ZNF135	0.05	0.073	2.01
ADA	0.00	0.000	3.28
BPY2B	0.04	0.288	2.28
C6orf105	0.02	0.103	3.38
C6orf218	0.01	0.147	2.48
CCL3L3	0.01	0.087	2.34
CD19	0.00	0.016	2.61
COG4	0.01	0.040	0.49
CYorf14	0.04	0.220	2.66
FAM72D	0.00	0.016	2.62
FER1L4	0.00	0.000	2.18
FGF9	0.00	0.036	2.21
FLJ33590	0.00	0.016	2.02
FLJ41170	0.00	0.052	2.75
HOXB7	0.04	0.103	2.03
MGC29506	0.01	0.000	2.07
NMS	0.03	0.326	2.15
PASD1	0.03	0.220	3.62
PPFIA2	0.03	0.312	3.05
PSCD4	0.01	0.000	2.04
STAP1	0.00	0.016	2.09
STS-1	0.01	0.000	2.55
TRAF3IP3	0.00	0.023	2.31
ZBP1	0.00	0.000	2.39
ZFY	0.02	0.120	2.49
EPS8L2	0.04	0.040	0.42
XPNPEP2	0.02	0.000	2.78
RRN3P2	0.00	0.000	2.29
SLC29A4	0.03	0.052	0.47

IC versus IT (46 DEG)	p-values	q-values	FC
ATG10	0.00	0.027	2.08
C16orf56	0.02	0.103	0.44
C17orf91	0.01	0.000	2.87
C20orf54	0.03	0.288	0.47
C5orf40	0.05	0.268	0.33
C8ORFK32	0.03	0.239	0.43
CCNA2	0.05	0.288	0.46
CD52	0.00	0.171	0.48
CITED1	0.02	0.171	0.29
CLRN1OS	0.00	0.040	0.44
DAND5	0.04	0.366	0.29
FAM19A3	0.04	0.326	0.45
FLJ43763	0.02	0.171	0.34
FLJ44653	0.04	0.288	0.26
FOXN1	0.02	0.129	0.28
HKDC1	0.00	0.288	0.28
KIR3DL1	0.04	0.312	0.26
LOC729234	0.01	0.087	0.46
LRFN2	0.03	0.288	0.42
MSI1	0.01	0.239	0.45
MUC20	0.01	0.120	2.13
MUCL1	0.05	0.268	0.48
MYT1	0.02	0.312	0.43
NMUR2	0.03	0.288	0.50
NPFFR1	0.03	0.129	0.30
NRSN1	0.00	0.220	0.37
OLIG3	0.05	0.312	0.32
OR1K1	0.03	0.171	0.30
PAK6	0.04	0.000	0.38
PDXDC2	0.03	0.000	2.63
PGA5	0.01	0.316	0.48
PYGM	0.03	0.366	3.02
SLC13A4	0.04	0.196	0.39
SLC22A12	0.04	0.196	0.42
SLC25A12	0.00	0.103	0.48
SLC8A2	0.03	0.349	0.44
SPHKAP	0.02	0.171	0.30
ST8SIA2	0.03	0.196	0.33
TSSK4	0.04	0.171	0.43
VWDE	0.04	0.052	0.46
ZMYND10	0.03	0.129	0.33
EPS8L2	0.03	0.040	0.39
XPNPEP2	0.01	0.000	2.18
BLK	0.01	0.000	0.47
RRN3P2	0.01	0.000	2.05
SLC29A4	0.01	0.052	0.35

ENEG versus IT (71 DEG)	p-values	q-values	FC
ATP8B3	0.04	0.196	2.40
C10orf90	0.04	0.381	2.43
CCDC142	0.01	0.087	0.50
CCR7	0.04	0.040	2.00
CENPK	0.02	0.065	2.03
CFHR3	0.01	0.103	0.49
CLCN4	0.03	0.196	2.03
COL9A2	0.02	0.057	2.17
DCPS	0.00	0.045	0.39
DLX6AS	0.05	0.381	2.64
DNAJB7	0.03	0.381	2.08
ERN2	0.02	0.196	2.22
FAM186B	0.02	0.087	2.12
FAM80B	0.04	0.147	2.55
FOXK2	0.04	0.103	0.50
FTO	0.00	0.000	0.42
GPR120	0.05	0.381	2.22
GSTM5	0.04	0.376	2.68
GZMH	0.00	0.040	2.53
HSD17B14	0.01	0.129	0.47
HSPB2	0.04	0.147	0.50
IL6	0.05	0.326	2.19
KHDC1	0.04	0.376	2.40
KIAA1202	0.02	0.057	0.49
MCTP2	0.00	0.045	2.53
MIR648	0.01	0.052	0.48
NCRNA00086	0.04	0.000	2.06
NRIP3	0.03	0.057	2.17
PPFIA4	0.05	0.000	2.06
PZP	0.04	0.171	0.46
RAB9B	0.03	0.000	2.43
RGAG1	0.02	0.171	0.35
RP1L1	0.03	0.326	2.43
SEMA3A	0.03	0.326	2.00
SEMA3F	0.01	0.045	0.47
SUCNR1	0.01	0.147	0.48
SYPL2	0.03	0.073	0.49
TBC1D22B	0.02	0.103	2.72
TKTL1	0.01	0.129	2.15
TMEM88	0.00	0.073	2.16
TMPRSS9	0.02	0.065	0.45
UBL4A	0.01	0.000	2.14
UPP2	0.02	0.000	0.28
ADA	0.00	0.000	2.26
BPY2B	0.02	0.000	2.81
C6orf105	0.03	0.103	2.80
C6orf218	0.02	0.000	2.90
CCL3L3	0.04	0.087	2.12
CD19	0.00	0.016	2.54
COG4	0.00	0.040	0.42
CYorf14	0.02	0.000	3.00
FAM72D	0.01	0.016	2.27
FER1L4	0.00	0.000	2.94
FGF9	0.00	0.036	2.33
FLJ33590	0.04	0.016	2.36
FLJ41170	0.02	0.052	2.38
HOXB7	0.02	0.103	2.14
MGC29506	0.00	0.000	3.01
NMS	0.02	0.326	2.29
PASD1	0.05	0.220	3.04
PPFIA2	0.03	0.000	4.23
PSCD4	0.02	0.000	2.00
STAP1	0.02	0.000	2.40
STS-1	0.00	0.000	2.08
TRAF3IP3	0.00	0.023	2.56
ZBP1	0.00	0.000	2.33
ZFY	0.00	0.120	3.18
BLK	0.00	0.000	0.35
RRN3P2	0.01	0.000	2.09
SLC29A4	0.05	0.052	0.43
