# Supplementary material for: Unique intrahepatic transcriptomics profiles discriminate the clinical phases of a chronic HBV infection
Source: PLoS One. 2017 Jun 29;12(6):e0179920. doi: 10.1371/journal.pone.0179920 (PMC5491066; doi:10.1371/journal.pone.0179920)
Supplement: S3 Table — (PDF) [file pone.0179920.s004.pdf]

S3 Table. The top varied modules with determined function were clustered into 2 clusters based on their transcriptional activity relative to the IT phase, which had distinct activity patterns in HBV clinical phases

*** ModuleID = the ID of transcriptomics modules developed by Chaussabel et al. Immunity 29, 150-164 (2008) (http://www.biir.net/public_wikis/module_annotation/V2_Trial_8_Modules), nr. of genes = number of genes included in the module, STDEV = Standard deviation of relative transcriptional activity of the module across all clinical phases, STDEV_Percentiles = the rank of the STDEV in the data set of all STDEVs of 260 modules, IFC = Intrinsic functional cluster, generated by unsupervised clustering of relative transcriptional activites.**

Module Group	ModuleID	Module Name	nr. of genes	IA vs IT	IC vs IT	ENEG vs IT	STDEV	STDEV_Percentiles	Gene Fold change > 1.5
Cluster1	M5.10	Mitochondrial Respiration	**237**	-3.10	2.91	-2.54	3.32	0.99	CCDC127;CISH;KIFAP3;NUDT2;PARN;WIBG
	M3.5	Cell Cycle	**151**	-2.64	2.89	-2.63	3.19	0.98	SAAL1
	M6.2	Mitochondrial Respiration	**158**	-3.10	2.71	-3.86	3.60	1.00	ARD1A;FBXL15;TBP
	M5.6	Mitochondrial Stress/Proteasome	**143**	-1.78	3.34	-3.43	3.53	0.99	XRCC6BP1
	M7.1	Inflammation	**184**	-0.17	3.17	-1.08	2.24	0.93	ACPP;FKSG30;GBGT1;IIP45;NCF2;PTPN6;RAB38;RIPK3;SH2B2;SLC16A5;ZNF213
	M5.1	Inflammation	**248**	-0.18	3.75	-1.52	2.74	0.95	BTBD10;GADD45G;GNA15;GNAI3;LOXL3;MAP3K8;RASSF3
	M4.5	Protein Synthesis	**89**	0.37	3.77	-1.78	2.80	0.95	NACAP1
Cluster2	M3.3	Cell Cycle	**50**	5.92	-2.08	2.61	4.02	1.00	APOBEC3B;ASPM;AURKA;C12orf48;CCNB2;CDC20;CDT1;GINS2;KIF11;NCAPG;RRM2;TIMD4;TPX2;UHRF1
	M4.10	B cell	**36**	4.04	-1.44	1.80	2.75	0.95	BLR1;BRDG1;C9orf45;CD19;EBF1;FCRLA;HLA-DOB;LOC90925
	M6.16	Cell Cycle	**32**	3.15	-1.83	0.80	2.49	0.96	APOBEC3H;CENPF;MCM2;SGOL1;UBE2T
	M6.11	Cell Cycle	**20**	0.88	-3.65	-0.14	2.38	0.94	CDC25A
	M8.46	Cytotoxic/NK	**19**	5.22	1.20	5.40	2.38	0.97	BTNL3;CXCR6;GZMB;KIR2DL4;KIR2DS5;KLRB1;PTGDR

Module Group	ModuleID	Module Name	nr. of genes	IA vs IT	IC vs IT	ENEG vs IT	STDEV	STDEV_Percentiles	Gene Fold change > 1.5
IFC1	M8.65	Undetermined	**12**	1.00	-1.75	1.93	1.91	0.94	GHRL
	M7.32	Undetermined	**15**	0.26	-1.73	1.51	1.64	0.92	TNFRSF13B
	M9.51	Undetermined	**12**	-1.00	-2.07	1.06	1.59	1.00	NPPA
	M8.71	Undetermined	**12**	1.82	-1.96	-0.40	1.90	0.93	MGC26718
IFC2	M7.27	Undetermined	**36**	0.29	3.05	0.05	1.67	0.91	RCVRN;TMEM88
	M9.16	Undetermined	**75**	0.15	3.28	-0.36	1.97	0.94	CCNYL1;CCRN4L;CYB5R2
	M5.5	Undetermined	**249**	0.42	4.83	-0.39	2.81	0.96	CAMSAP1;DDX10;LOC92017;SAMM50;TEX264;TRAF3IP3;ZNF211;ZNF614
	M7.3	Undetermined	**173**	0.17	4.29	-1.15	2.84	0.98	
	M5.8	Undetermined	**113**	1.49	5.62	-0.37	3.07	0.98	AOF2;AP4B1;C17orf68;EIF2B5;GPD1L;TIMM22;TTF2
	M7.25	Undetermined	**106**	1.28	3.77	-0.31	2.06	0.96	C17orf68;NAT9;TMC6;TUBG2;WDR73
	M4.12	Undetermined	**95**	1.79	4.93	0.21	2.40	0.91	ALKBH3;NXT1;PCID2
IFC3	M5.4	Undetermined	**132**	-2.49	3.92	-2.46	3.69	1.00	
	M9.15	Undetermined	**78**	-0.33	3.49	-1.18	2.49	1.00	C18orf26;C6orf81;JAG1;SYCP2
	M8.6	Undetermined	**37**	-0.62	2.43	-1.41	2.03	0.96	ZNF589;ZNF761
	M8.2	Undetermined	**51**	-1.53	2.62	-1.24	2.32	0.98	
	M5.13	Undetermined	**182**	-2.57	3.90	-3.40	4.00	1.00	ANKRD37;CD48;COMMD2
	M7.11	Undetermined	**134**	-1.01	3.94	-1.32	2.95	0.99	DNASE2;NFKBIE;OBFC1;P15RS;ZFY
	M7.19	Undetermined	**99**	-0.93	4.18	-1.60	3.16	1.00	EXOSC7
	M9.8	Undetermined	**100**	-0.37	3.59	-1.65	2.73	1.00	CGRRF1;FKBPL;GZF1;SPATA2
	M9.28	Undetermined	**47**	-0.78	2.62	-1.07	2.05	1.00	SMC6
	M7.20	Undetermined	**48**	-1.10	2.52	-1.82	2.32	0.97	ANP32C;SNRPE
	M7.5	Undetermined	**156**	-0.99	2.84	-1.55	2.39	0.96	ANKRD13C;COMMD9;ZFP90;ZNF263;ZSCAN2
	M6.7	Undetermined	**76**	-0.02	3.45	-1.84	2.69	0.97	LPXN;LRRC8C
	M7.9	Undetermined	**167**	-0.63	2.35	-1.33	1.96	0.92	FUT4
	M7.8	Undetermined	**112**	-1.88	2.50	-3.45	3.08	0.99	
	M7.6	Undetermined	**104**	-0.04	3.37	-1.38	2.45	0.96	KCNA3;MCM5;NOLC1;SH3PXD2A
	M7.4	Undetermined	**140**	-0.96	2.85	-2.04	2.57	0.97	C3orf21;RFWD3;TMEM138;UCHL5IP
IFC4	M7.30	Undetermined	**72**	-3.62	1.43	-2.40	2.63	0.99	RNPC3;SAP30
	M9.21	Undetermined	**66**	-2.26	0.47	-2.01	1.51	0.94	MYCBP;P2RY12;VEPH1
	M9.3	Undetermined	**173**	-3.56	1.01	-4.06	2.79	1.00	CD300LB;CEP164;COL9A2;MYL5;NDST2;PNMA6A;PPFIA4;SMPD2;WDR25
	M9.52	Undetermined	**11**	-1.67	-0.20	-1.89	0.92	1.00	
	M6.10	Undetermined	**79**	-3.05	1.50	-3.32	2.71	0.97	
	M7.28	Undetermined	**71**	-1.50	1.77	-1.80	1.98	0.95	
	M9.23	Undetermined	**57**	-1.29	0.69	-1.67	1.27	0.93	C20orf103;CCL3L1
	M9.20	Undetermined	**66**	-1.88	1.61	-2.25	2.13	1.00	GPR174
	M9.17	Undetermined	**75**	-1.13	1.63	-1.93	1.87	0.94	CDC25C;ZNF341
	M8.3	Undetermined	**48**	-1.60	0.70	-2.51	1.65	0.93	RPUSD1
	M8.84	Undetermined	**10**	-1.10	1.12	-2.37	1.77	0.91	
